# Utilizing industrial byproducts for the manufacture of clay-cellulose nanocomposite cements with enhanced sustainability

**DOI:** 10.1038/s41598-023-51130-z

**Published:** 2024-01-07

**Authors:** M. S. El-Feky, Amr H. Badawy, Passant Youssef, Mohamed Kohail

**Affiliations:** 1 Department of Civil Engineering, National Research Centre, Giza, Egypt; 2 Structural Engineering Department, German University in Cairo, Cairo, Egypt; 3 Structural Engineering Department, Ain Shams University, Cairo, Egypt

**Keywords:** Civil engineering, Nanoscale materials

## Abstract

This study investigates the influence of different nano clay contents (0, 1, 3, and 5 wt% of cement) on the microstructure and the mechanical properties of cement composites reinforced with varying Nano cellulose fiber contents (0, 0.5, 0.75, and 1 wt% of cement). Unlike previous research that employed sonication to improve dispersion in the cement matrix, this study explores the effects of unsonicated nano-cellulose addition and the combined incorporation of nano-cellulose and nano-clay. The results demonstrate that these additions significantly enhance the compressive strength, abrasion resistance, and water absorption ratios of the cement composites. Furthermore, the inclusion of nano-clay improves the microstructure of the cement matrix, strengthening the interfacial transition zone and reinforcing the bond between nano-cellulose and the cement matrix. The microstructural analysis using scanning electron microscopy (SEM) reveals the presence of a dense interconnected structure characterized by rod-like crystals. This research contributes to the development of sustainable construction materials by examining the effects of nano-cellulose and nano-clay on the properties and microstructure of cement composites. The utilization of industrial byproducts, such as wood sawdust, for the extraction of nano-cellulose offers an eco-friendly approach to enhance the performance of cement-based materials. The maximum compressive strength obtained, after 28 days, was at mix with 0.75% NCL + 5%NC with a gain of 53.5% than that of the control mix. In mixes containing only nano-clay (NCL), the increase in NCL content led to a higher rate of water absorption in the cement matrix, which reaches 4%. Confirming the results obtained from compressive strength and water absorption, mix with 0.75% NCL and 5% NC had obtained the optimum values with an improvement of 20% than that of the control mix.

## Introduction

The durability of concrete structures has become a significant focus of research in recent years. Various approaches have been explored to enhance the durability of concrete, including the use of supplementary cementitious materials (SCMs), fibers, and nanomaterials. Industrial waste materials can serve as both SCMs and nanomaterials, offering a potential partial replacement for cement. Fly ash (FA)^1–3^, silica fume (SF)^4–8^, ground-granulated blast furnace slag (GGBS)^9–12^, and metakaolin (MK)^13–15^ have been investigated as micro SCMs for cement replacement. Additionally, nano-silica (NS)^16–25^, nano-clay (NC)^26–30^, carbon nanotubes (CNT)^31–35^, nano-cellulose (NCL)^36–40^, and nano-titanium^41–43^ have recently garnered attention as partial replacements for cement in concrete and cement mortars. The utilization of eco-friendly SCMs as partial cement replacements can help mitigate the environmental impact of the cement industry^44–46^. Incorporating different nanomaterials has shown potential to enhance the durability of concrete structures, prolong their service life, and reduce maintenance costs^47^.

Another approach to improve the mechanical and durability properties of concrete is the use of various types of fibers^48–51^. However, the high cost, low bond strength, and limited corrosion resistance of fibers have hindered the fibers’ widespread use in cement composites. Carbon and polymer fibers, for instance, are relatively expensive compared to other concrete ingredients, leading to higher concrete costs^52^. Glass fibers, on the other hand, exhibit low bond strength with cement and poor alkaline resistance^53,54^. To overcome these limitations, researchers have explored the potential of nano-cellulose as a nano fiber reinforcement for concrete and cement paste^55,56^. Nano-cellulose can be derived from plants, animals, and bacteria, and it offers advantages such as stiffness, lightweight nature, availability, environmental friendliness, and surface roughness that improves its bond with cement composites^57–59^. Moreover, nano-cellulose has been found to enhance concrete's flexural strength, toughness, and impact resistance^60–62^. While nano-cellulose has shown improvements in flexural strength of cement composites by 20–50%, its effect on compressive strength remains a topic of investigation^63–67^. Consequently, further studies are needed to determine the optimal dosage of nano-cellulose^68^ and explore its implementation with different cement composites^69^.

In this research, we propose the addition of Nano-Clay to nano-cellulose mixes to enhance the homogeneity of the cement paste and improve compressive strength, while nano-cellulose focuses on enhancing flexural and tensile strength. The effect of nano-clay on nano-cellulose mixes will be investigated through compressive strength, water absorption, and abrasion tests. Additionally, scanning electron microscopy (SEM) and thermogravimetric analysis (TGA) will be employed to study the impact of nano-cellulose and nano-clay on the microstructure of the cement matrix.

## Experimental program

### Materials

**Figure 1:**
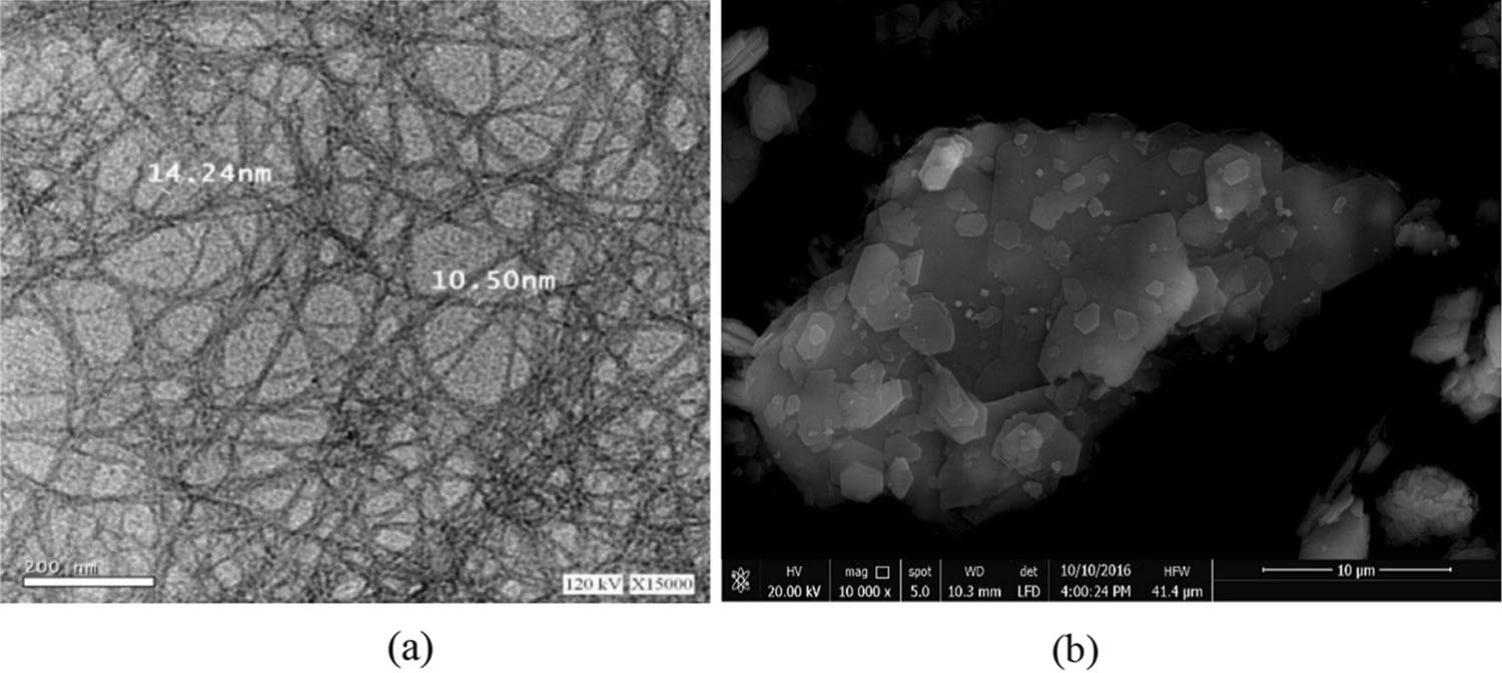
SEM micrographs of (**a**) Nano-cellulose particles, (**b**) Nano-Clay.

In this experimental program, ordinary Portland cement was utilized, conforming to ASTM C150 standards^70^, with a grade of 42.5N. The used natural clean sand adhered to ASTM C33 standards^71^. The Nano-Clay utilized in this study was sourced from the Egyptian market and underwent calcination of kaolinite to achieve an off-white color with an average particle size of 30 nm. The chemical compositions of the cement and Nano-Clay used in this study are provided in Table 1. Nano-cellulose, obtained from NCTECH company, had a diameter ranging between 5 and 11 nm and a length of 120–400 nm. The nano-cellulose was extracted from wood sawdust, and the production process effectively removed impurities such as lignin, pectin, wax, and soluble sugars. The nano-cellulose was introduced as a fully dispersed gel, with a concentration of 5% nano-cellulose. Figure 1 showcases the scanning electron microscopy (SEM) images of the nano-cellulose and metakaolin employed in this experimental program. A Naphthalin sulphonate-based superplasticizer of type F was utilized in the experimental program.

**Table 1:** Chemical composition of cement, and nano-clay (%).

	SiO_2_	Fe_2_O_3_	Al_2_O_3_	CaO	MgO	TiO_2_	Na_2_O	K_2_O	P_2_O	L.O.I
Cement	20.13	3.61	5.32	61.63	2.39	–	0.37	0.13	–	1.96
MK	61.24	1.06	20.89	0.16	0.22	1.61	0.71	–	–	14.11

### Sampling

A total of 28 mixes were prepared, resulting in 192 specimens. To investigate the influence of nano-cellulose as the sole additive to the cement mortar, three mixes were formulated with addition ratios of 0.5, 0.75, and 1 wt% of cement. Similarly, three mixes were prepared with Nano-Clay at addition ratios of 1, 3, and 5 wt% of cement. Additionally, to study the combined effect of Metakaolin and nano-cellulose, nine mixes were designed. These included three mixes with 0.5 wt% of nano-cellulose and 1, 3, and 5 wt% of Nano-Clay, as well as three mixes with 0.75 wt% of nano-cellulose and the same addition ratios of Nano-Clay. Similarly, three mixes with 1 wt% of nano-cellulose and the corresponding addition ratios of Nano-Clay were prepared. The mix proportions of the designed mixes are presented in Table 2. The control mix, which served as a reference, did not contain any additive, whether nano-clay or nano-cellulose.

**Table 2:** Mix proportion of cement mortars with nano-cellulose and nano-clay.

Mix	Sand (gm)	Cement (gm)	NCL (%)	NC (%)	Water (%)	S.P (%)
0%NCL + 0%NC	3000	1000	0	0	45	2
0.5%NCL + 0%NC	3000	1000	0.5	0	45	2
0.75%NCL + 0%NC	3000	1000	0.75	0	45	2
1.0%NCL + 0%NC	3000	1000	1	0	45	2
0%NCL + 1%NC	3000	1000	0	1	45	2
0%NCL + 3%NC	3000	1000	0	3	45	2
0%NCL + 5%NC	3000	1000	0	5	45	2
0.5%NCL + 1%NC	3000	1000	0.5	1	45	2
0.5%NCL + 3%NC	3000	1000	0.5	3	45	2
0.5%NCL + 5%NC	3000	1000	0.5	5	45	2
0.75%NCL + 1%NC	3000	1000	0.75	1	45	2
0.75%NCL + 3%NC	3000	1000	0.75	3	45	2
0.75%NCL + 5%NC	3000	1000	0.75	5	45	2
1%NCL + 1%NC	3000	1000	1	1	45	2
1%NCL + 3%NC	3000	1000	1	3	45	2
1%NCL + 5%NC	3000	1000	1	5	45	2

In the mixing process, nano-cellulose and/or Nano-clay were initially added to half of the mixing water and thoroughly mixed together. Subsequently, this mixture was added to the dry mix comprising cement and sand. After adding the first half of the mixing water with the nano-materials, the mortar mixer was stirred for 2 min. Then, the second half of the mixing water, combined with the superplasticizer, was added to the mortar mixer, and the mixture was stirred for an additional 3 min. After the completion of the mixing process, the mortar was poured into molds. To guarantee thorough compaction of the specimens, the molds were positioned on a vibrating platform. Following casting, the specimens were left undisturbed for a duration of 2 h to permit the cement mortars to achieve their final setting. Subsequently, the specimens underwent water curing at a constant temperature of 25 ºC until the day of testing, ensuring optimal curing conditions for the assessment.

### Test methods

The average compressive strength results were obtained by taking measurements from three samples measuring 5 × 5 × 5 cm for each mix. The testing was performed using a SHIMADZU 1000 KN universal testing machine, with a loading rate of 0.5 N/mm2 applied after 7 and 28 days of curing. The test procedure adhered to the guidelines specified in the ASTMC109-C109M-20 Standard^72^.

To assess the durability of the hardened mixes, a water absorption test was performed on 5 × 5 × 5 cm cubes. The cubes were submerged in water for 24 h and then dried in an oven at 100 °C for another 24 h. The weight of water absorbed by the cubes was calculated. This test was conducted according to the ASTM C642-21^73^ standard.

For the abrasion test, 7 × 7 × 7 cm cubes were subjected to abrasion. The cubes were placed on a grinding path on the abrasion machine and sprayed with 20 gm of standard abrasion powder (sand). The weight loss was then calculated. The abrasion test on cement mortar followed the ASTM C944 standards^74^. The water absorption test and the abrasion test were conducted following a 28-day curing period. For each test, three specimens were prepared, and the average outcome was documented.

The compaction and curing procedures for all specimens adhered to the guidelines specified in ASTM C31^75^.

To analyze the microstructural properties of the samples, Scanning Electron Microscopy (SEM) and Thermogravimetric Analysis (TGA) techniques were employed. The SEM analysis was conducted using a QUANTA FEG250 instrument on crushed cement mortar cubes. Secondary electron images were obtained from samples coated with Au and using a voltage of 20 kV. This analysis aimed to determine the size and distribution of nano-particles, characterize the cement paste mixtures, and aid in interpreting the compressive strength results of the samples after 28 days of water curing.

Thermogravimetric Analysis (TGA) was employed to assess the effect of high temperatures on hydrated cement composites. A TGA850 thermo balance from Mettler Toledo, along with STARe software v8.10, was utilized. The samples were milled, washed with acetone, filtered, and then dried at 60 ± 2 °C for approximately 30 min. Aluminum crucibles with a capacity of 100 mL were filled with 30 ± 1 mg of the dried sample. The samples were heated up to 1000 °C at a heating rate of 10 °C/min under a nitrogen atmosphere.

## Experimental results

### Compressive strength

**Figure 2:**
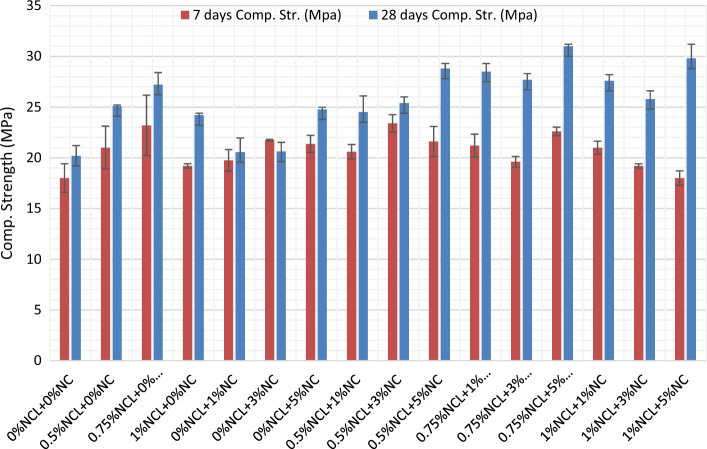
7 and 28-day compressive strength of NCL and NC cement mortar mixes.

**Figure 3:**
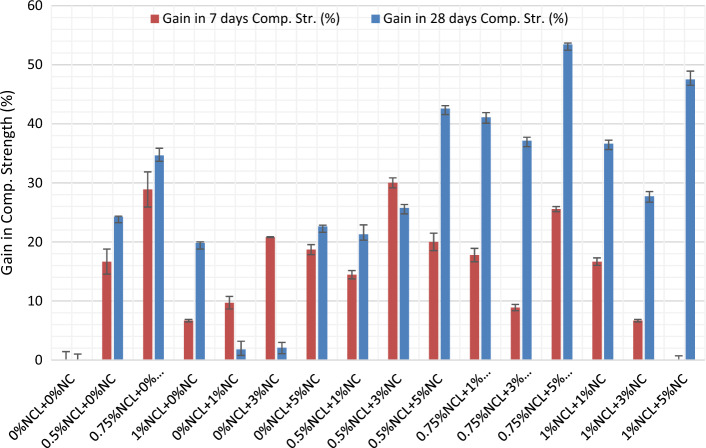
Gain in compressive strength at 7 and 28-day for NCL and NC cement mortar mixes.

The compressive strength results for the cement paste mixes after 7 and 28 days and their gain in strength than the control mix are shown in Figs. 2 and 3, respectively. From the figures below, NCL and NC had improved the compressive strength of the cement mortars generally with a difference percentage individually and combined with different ratios, except for mixes 0%NCL + 1%NC and 0%NCL + 3%NC. However, after 7 days the strength was gained by 10% and 21%, respectively. This can be due to the low replacing ratio of NC in the mix as it is not enough for gaining strength, besides to the agglomeration occurred to NC in the cement matrix due to the improper dispersion. While at the same percentages of NC when adding NCL, the strength was improved as in the mix.

The maximum compressive strength obtained, after 28 days, was at mix with 0.75% NCL + 5%NC with a gain of 53.5% than that of the control mix. This is clear that, at using 0.75% of the cement weight with nano-cellulose gives the highest strength and this is clear in mixes containing NCL only; as at 0.75% NCL the gain in strength was 35% and when increasing the content of NCL to 1% of the cement weight, the strength gained reduced to 20% than of the control mix. This can be attributed to the that using adequate replacement ratios of NCL (0.75%) can be more efficient to the pozzolanic reaction than using lower or higher replacement ratios as done in many types of research in which it reached 5% and 10% as a replacement ratio^76–78^; resulting in a reaction with the produced calcium hydroxide and formation more C–S–H, besides the densification for the interfacial transition zone (ITZ) between the aggregates and the cement paste^79–81^. The relatively lower strength gain observed in NCL (nano-clay) mixes after 7 days can be attributed to the presence of hydroxyl and carboxyl groups in cellulose. These functional groups react with calcium ions (Ca+) to form complexes, which negatively affect the hydration reaction and contribute to a delayed setting time^82^. However, after 28 days, the water absorbed by NCL is released into the cement matrix. This release plays a significant role in the continuous hydration of unreacted cement particles, leading to improvements in the mechanical properties and microstructure of NCL mixes^83,84^ The same when studying the effect of NC on the strength, by increasing NC content from 1 to 5% of the cement weight the strength gain increased from 2 to 23%. However, the lower compressive strength after 7 days of NC mixes was due to the relatively high specific surface area of NC, which absorbs more water than reducing the amount of hydrated cement. Besides, Nano-Clay used was not subjected to the sonication process besides the relatively high replacement ratio, which reaches 5% by weight of cement. In addition, the dilution effect of Portland cement increases by increasing Nano-Clay content in the mix^14^. While after 28 days, the compressive strength of NC mixes had improved by a narrow limit. This can be attributed to the pozzolanic effect of NC, which increases the pozzolanic products CSH, CASH, and CAH, and the trapped water had released to improve the hydration process but with a narrow limit^85,86^.

When investigating the combined effect of NCL with NC, generally, the addition of NCL has a positive effect on the strength of NC mixes especially at the late ages; however, at 7 days at specific mixes, an improvement had occurred with negligible percentages as reported by^87^. Adding NCL particles to NC mixes improves the compressive strength of the mixes, with an improvement reaching 24% more than that without NCL. This can be explained by the needle effect or the fiber effect of NC particles that combine with cellulose nano-fibers to reduce the NC mixes' cracks^18,26^. Besides the reactivity and the nucleation effect of NC, the fiber effect of NC has been improved in much literature to help enhance the compressive strength and the other mechanical properties^28,88,89^. However, using NC without sonication leads to agglomeration of NC particles, and thus a reduction in the strength had occurred as shown in NC mixes only. While adding NCL to NC mixes, extra nano-fibers enhanced the strength of the mixes regardless of their dispersion in the matrix, resulting in an improvement in the packing density of the mix. This fiber effect fights against the dilution effect of cement and also against the agglomeration of NCL and NC particles.

### Water absorption test

Water absorption in cement matrices is primarily influenced by the capillary action of the matrix. The findings of the water absorption test exhibited a consistent pattern with the results obtained for compressive strength. As the compressive strength increased, water absorption decreased. This correlation suggests that the factors influencing the strength of the mixtures also contribute to the reduction of water absorption properties. The decrease in water absorption can be attributed to the filling effect of clay nano-particles and the flask-shaped structure of these particles, which provide damping effects. Moreover, the pozzolanic reactivity of nano-cellulose (NC) improves the microstructure of the cement matrix by reducing voids and porosity, as previously mentioned in^26,28,90–92^.

**Figure 4:**
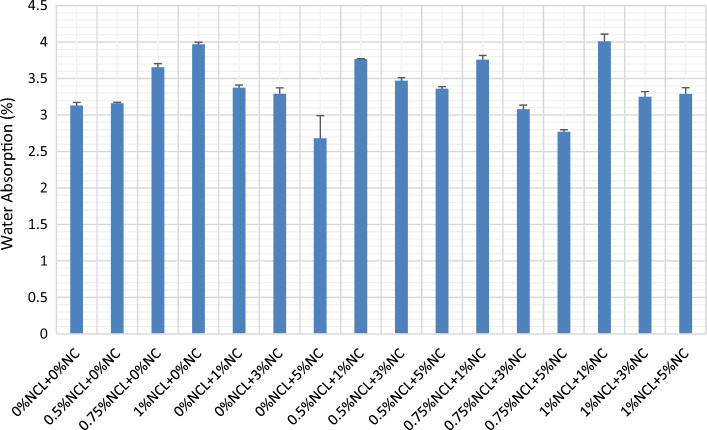
Water absorption of NCL and NC cement mortar mixes.

In mixes containing only nano-clay (NCL), the increase in NCL content led to a higher rate of water absorption in the cement matrix, as depicted in Fig. 4. This outcome can be attributed to improper dispersion and agglomeration that occurs when using high concentrations of cellulose nano-fibers. Similar trends were observed in the compressive strength results, where increased water absorption was linked to inadequate dispersion and subsequent agglomeration. This weakens the bond between cellulose nanofibers and the cement matrix, resulting in the formation of voids and an increase in the porosity of the mixture. Consequently, water absorption is enhanced^93,94^.

### Abrasion test

**Figure 5:**
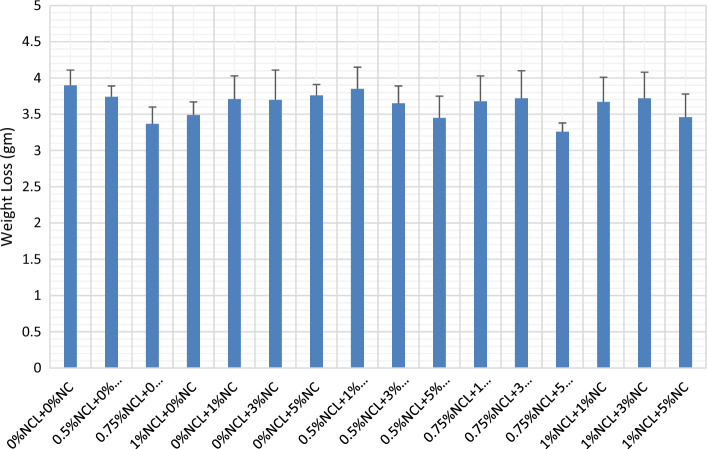
Weight loss in NCL and NC cement mortar mixes.

Adding nano-clay with cellulose nano-fibers had improved the cement mortar abrasion resistance, whether by using each one alone or through a combination of them, as shown in Fig. 5. Nano-clay had improved the homogeneity of the cement matrix through the pozzolanic and nucleation effect of nano-clay in the cement matrix^26,28^. Confirming the results obtained from compressive strength and water absorption, mix with 0.75% NCL and 5% NC had obtained the optimum values with an improvement of 20% than that of the control mix. this can be attributed to proper dispersion of NCL and NC in the cement matrix, in which cellulose nano-fibers improve the tensile strength of the cement matrix besides improving the crack propagation. While nano-clay had improved the microstructure of the cement matrix through filling, nucleation, pozzolanic and damping effect, in addition to the needle effect of clay nano-fibers as stated before in^26,28,30,37,79^.

### Microstructure analysis

#### Scanning electron microscope (SEM)

**Figure 6:**
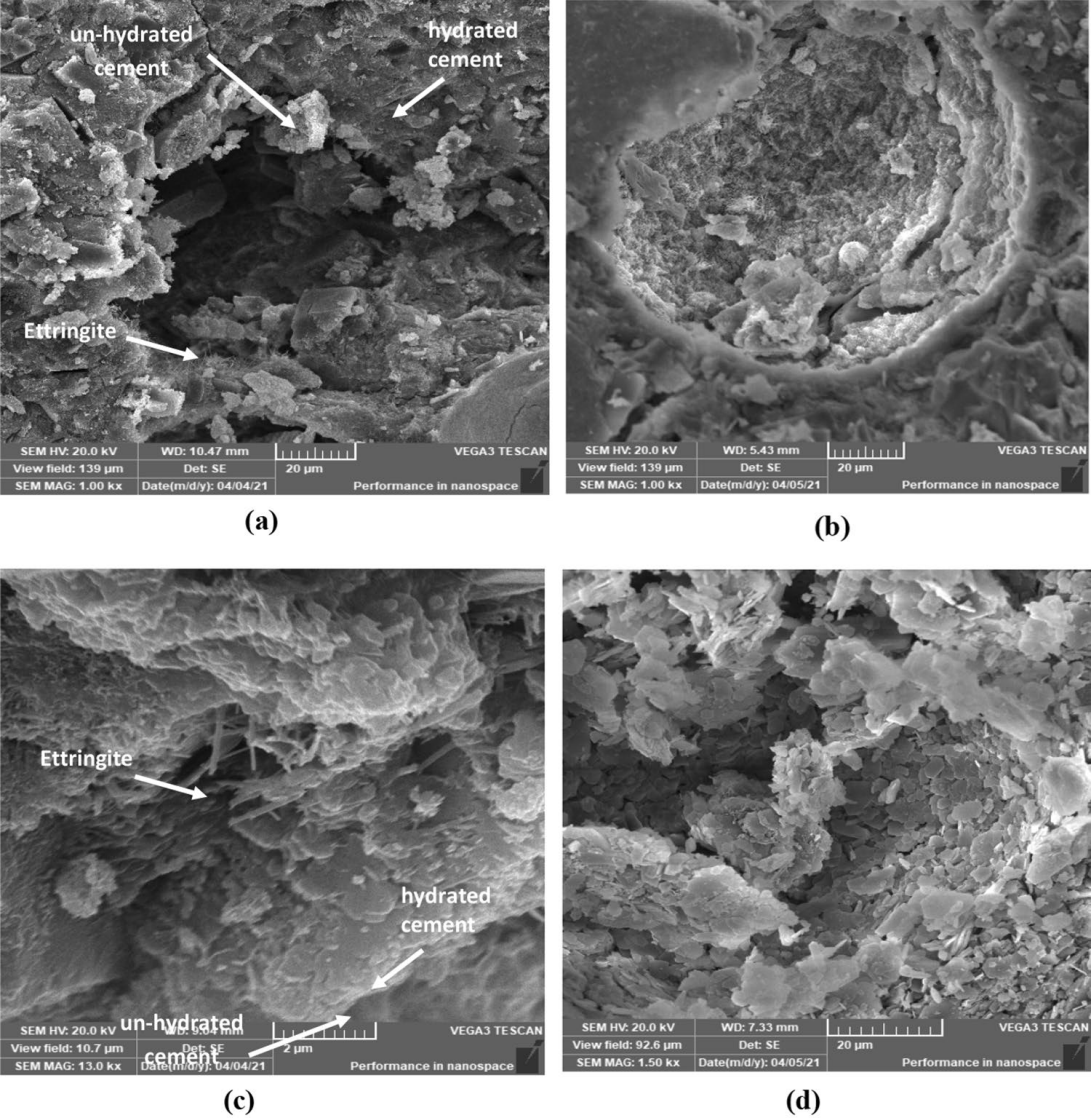
Scanning electron micrograph (SEM) of (**a**) specimen with 0% NCL and 0% NC; (**b**) specimen with 0% NCL and 5% NC; (**c**) specimen with 1% NCL and 0% NC; (d) specimen with 0.5% NCL and 3% NC.

The addition of nano-cellulose (NC) and nano-clay (NCL) had a significant impact on the microstructure of the cement matrix, as observed in Fig. 6b–d, compared to the mix without nanomaterials shown in Fig. 6a.

In the mix without nano materials (Fig. 6a), unhydrated calcium silicate hydrate (CSH) and calcium hydroxide crystals were observed in the form of small grains, along with ettringite needles and relatively large voids, indicating a less dense and weaker matrix.

In the matrix incorporating nano-clay particles (Fig. 6b), the presence of nano-clay contributed to densification of the matrix. The SEM micrograph revealed CSH products spreading over calcium hydroxide crystals, with clay nano-fibers dispersed throughout the matrix. This filling and nucleating effect of nano-clay helped to improve the microstructure and enhance the strength of the matrix.

Figure 6c shows the microstructure of the mix with 0% nano-clay and 5% nano-cellulose. In this case, nano-cellulose fibers were clearly visible, reinforcing the CSH products and significantly enhancing the matrix strength, as mentioned in relation to the compressive strength results. The presence of nano-cellulose also led to improved pore porosity and size, which can be attributed to its ability to absorb water and promote cement hydration, resulting in the formation of more cement hydrates.

In the case of the mixture containing 0% nano-clay and 3% nano-cellulose (Fig. 6d), the distribution of calcium silicate hydrate (CSH) products was observed, while the presence of calcium hydroxide crystals was minimal. This observation suggests that nano-cellulose exhibits a high reactivity with residual calcium hydroxide, resulting in a more compact and rigid matrix. Moreover, nano-clay was found to act as a bridge between the CSH products, effectively impeding crack propagation in its early stages. This finding highlights the effectiveness of the hybrid nanomaterials in enhancing the strength and durability of the matrix by reducing both the width and depth of cracks.

#### TGA

**Figure 7:**
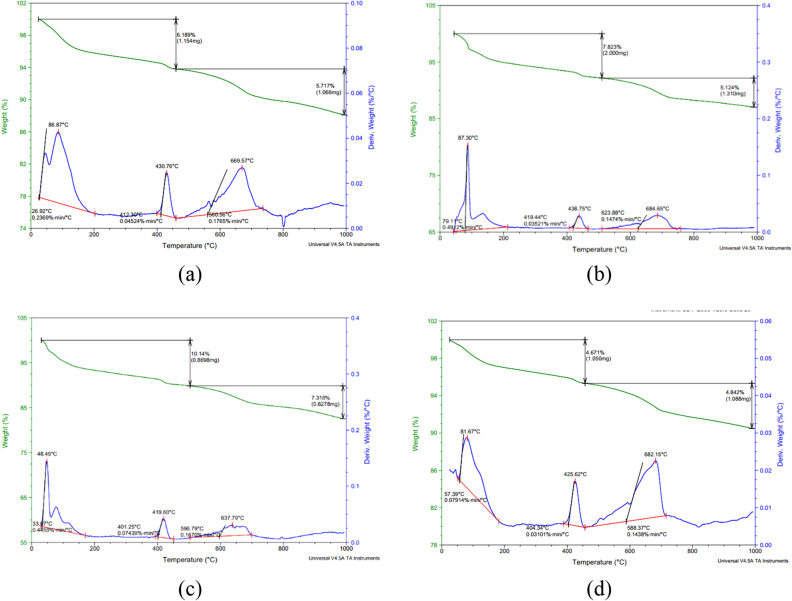
TGA results for (**a**) control specimen; (**b**) specimen with 0% NCL and 5.0% NC; (**c**) specimen with 1% NCL and 0% NC; (**d**) specimen with 1% NCL and 5.0% NC.

This study employed thermogravimetric analysis (TGA) to characterize the influence of nano-modifications on cement hydration behavior and product assemblages. Weight loss curves from TGA scans in Fig. 7 revealed differences in calcium hydroxide (CH) decomposition between 400 and 450°C^95,96^. The control paste exhibited a 6.1% loss, while mixes with nano-clay, nano-cellulose, and their hybrid showed 7.8%, 10.14%, and 4.6% reductions, respectively.

The weight loss observed within the temperature range of 400 to 450 degrees in the hybrid nano-material matrix indicates a relatively low amount of calcium hydroxide (CH), reaching only 4.6% loss by weight of the CH content. This finding suggests the limited presence of CH and consequently implies an increase in the formation of calcium silicate hydrate (CSH). This increase can be attributed to the pozzolanic reactivity of the nano clay particles. Additionally, water trapped within the nano-cellulose structure was released at later ages, driving further hydration of unreacted cement grains.

Conversely, the nano-cellulose only mix featured the greatest CH loss (10.14% compared to 6.1% for the control mix), likely due to its nano-scale dimensions serving as active nucleation sites enhancing hydration kinetics. This promoted development of larger, more completely formed CH crystals over time. The substantial surface area of nano-cellulose may have also interfered with cement particle dissolution through adsorption interactions, temporarily delaying hydration.

Overall, TGA provided meaningful insight into the mechanisms by which various nano-additives alter cement hydration. Nano-clay most efficiently consumed CH due to its pozzolanic behavior. Conversely, nano-cellulose actively promoted hydration but interacted strongly with cement, delaying dissolution. The hybrid system balanced these effects, yielding optimal hydration and the lowest residual CH content indicative of advanced hydration. Careful nano-material selection and combination is thus crucial to fully capitalize on their hydration modifying abilities.

Upon comparing the outcomes of this study with the findings of El-Tair et al.^62^, several significant distinctions are evident. In their research, the addition of nano-clay and cellulose nano-fibers to the mixture took place subsequent to sonication, and the dosages employed were higher in comparison to the present investigation. The outcomes of their study demonstrated improvements in the mechanical and durability properties of the resulting cement composites.

In contrast, the present research incorporated nano-clay and cellulose nano-fibers at smaller percentages without sonication. This difference in dosage and the absence of sonication may have influenced the overall performance of the nanomaterials in the cement matrix. Sonication plays a crucial role in enhancing the dispersion of nano-materials and achieving a homogeneous microstructure within the matrix. Therefore, the lack of sonication in the current research may have limited the effectiveness of the nano-clay and nano-cellulose in improving the properties of cement composites^97–100^.

It is worth mentioning that another study conducted by El-Feky et al.^26^ also employed sonication prior to the addition of nano-cellulose and nano-clay to the mixture. This further emphasizes the importance of sonication in optimizing the performance of nanomaterials within the cement matrix. The sonication process helps to enhance the dispersion of the nanoparticles and ensures a more uniform distribution throughout the matrix, leading to improved mechanical and durability properties.

Overall, the differences in dosage and the absence of sonication in the current research might explain the variation in results compared to previous studies. Sonication is a critical step in achieving the desired dispersion and microstructure for nano-materials within cement composites, and its incorporation can significantly enhance their performance.

## Conclusions

- The hybridization of nano-clay and nano-cellulose resulted in improved mechanical properties by enhancing stability, uniformity, and mix performance. It also led to improved homogeneity and compactness of the hardened matrices.
- At lower concentrations, the effectiveness of nano-cellulose was more pronounced. This can be attributed to its larger surface area, which decreases the likelihood of agglomeration, encourages self-assembly of fibers, and supports the formation of a network structure. These factors collectively enhance the mechanical properties and augment the resistance to crack propagation.
- Microstructure analysis revealed that the addition of small quantities of nano-clay improved its dispersion, homogeneity within the cement matrix, and enhanced pozzolanic reactivity.
- The pore distribution analysis demonstrated that the incorporation of both nano-clay and nano-cellulose improved the microstructure of the cement matrix.
- The addition of nano-cellulose had a positive effect on promoting the hydration of cement particles and enhancing the resistance of the cement matrix against cracking.

In summary, this study provides insights into the comparative effects of different nano-material addition methods on the mechanical properties and microstructure of cement composites. The findings highlight the importance of sonication in achieving improved dispersion and homogeneous microstructures. Furthermore, the results demonstrate the potential of nano-clay and nano-cellulose as promising additives for enhancing the performance of cementitious materials.

It is highly recommend conducting a comprehensive set of durability tests, including chloride diffusion, sulfate attack, and carbonation resistance, for future work. These tests will provide valuable insights into the performance and longevity of nano-modified mixes. Additionally, it is crucial to assess the flowability, rheology, and workability of these mixes through precise measurements. By evaluating these parameters, you can ensure that the nano-modified mixes possess the desired properties and can be effectively utilized in various construction applications. This information will not only contribute to the advancement of the field but also guide the selection and optimization of nano-modified mixes for enhanced performance and durability.

## Data Availability

All data generated or analyzed during this study are included in this published article.
